# Comprehensive analysis of NAC transcription factors and their expression during fruit spine development in cucumber (*Cucumis sativus* L.)

**DOI:** 10.1038/s41438-018-0036-z

**Published:** 2018-06-01

**Authors:** Xingwang Liu, Ting wang, Ezra Bartholomew, Kezia Black, Mingming Dong, Yaqi Zhang, Sen Yang, Yanling Cai, Shudan Xue, Yiqun Weng, Huazhong Ren

**Affiliations:** 1 Beijing Key Laboratory of Growth and Developmental Regulation for Protected Vegetable Crops, College of Horticulture, China Agricultural University, 100193 Beijing, P. R. China; 2 Department of Horticulture, USDA-ARS, Vegetable Crops Research Unit, University of Wisconsin-, Madison, WI 53706 USA

## Abstract

The cucumber (*Cucumis sativus* L.) is an important vegetable crop worldwide, and fruit trichomes or spines are an important trait for external fruit quality. The mechanisms underlying spine formation are not well understood, but the plant-specific NAC family of transcription factors may play important roles in fruit spine initiation and development. In this study, we conducted a genome-wide survey and identified 91 *NAC* gene homologs in the cucumber genome. Clustering analysis classified these genes into six subfamilies; each contained a varying number of *NAC* family members with a similar intron–exon structure and conserved motifs. Quantitative real-time PCR analysis revealed tissue-specific expression patterns of these genes, including 10 and 12 that exhibited preferential expression in the stem and fruit, respectively. Thirteen of the 91 *NAC* genes showed higher expression in the wild-type plant than in its near-isogenic trichome mutant, suggesting their important roles in fruit spine development. Exogenous application of four plant hormones promoted spine formation and increased spine density on the cucumber fruits; several *NAC* genes showed differential expression over time in response to phytohormone treatments on cucumber fruit, implying their essential roles in fruit-trichome development. Among the *NAC* genes identified, 12 were found to be targets of 13 known cucumber micro-RNAs. Collectively, these findings provide a useful resource for further analysis of the interactions between *NAC* genes and genes underlying trichome organogenesis and development during fruit spine development in cucumber.

## Cucumber cultivars: Genes could offer a spiny solution

A group of genes connected with spine growth in cucumber fruits could enable the development of cultivars for specific markets. In China, people prefer cucumbers with plentiful spines, while for the European market, cucumbers are usually smooth. This has implications for growers, yet the molecular mechanisms inherent in spine or 'trichome' growth are not completely clear. Yiqun Weng at the University of Wisconsin-Madison in the United States, together with Huazhong Ren at the China Agricultural University in Beijing, and co-workers identified genes related to trichomes in cucumbers. In a genome-wide survey, the researchers identified 91 genes from a family of transcription factors predicted to influence trichome growth. Of these, six genes were highly expressed in wild-type spiny cucumbers, while a further three were found highly expressed in mutant smooth cucumbers, indicating that they may reduce spine growth.

## Introduction

Transcription regulation of gene expression is an important control point of many biological processes in plant growth and development, as well as responses to biotic and abiotic stresses^1–3^. During this process, transcription factors (TFs) function as regulatory *trans*-acting elements that bind to specific *cis*-regulatory elements in the promoters of target genes to activate or repress expression of the target genes^4^. Thus, the identification and functional characterization of TFs is of great importance in understanding the transcriptional regulatory networks controlling different cellular processes^5^. Numerous plant TF families have been identified and characterized according to their DNA-binding motifs. Some notable examples include apetalous (AP2), basic leucine zipper domain (bZIP), myeloblastosis oncogene (MYB), myelocytomatosis oncogene (MYC), Mcm1-Agamous-Deficiens-Srf (MADS), homeodomain leucine zipper (HD-Zip), basic helix–loop–helix (bHLH), Cys2/His2-type zinc finger (C2H2), as well as the NAC (NAM, ATAF1/2, and CUC) transcription factor families^6–8^. The NAC is among the largest plant-specific TF families. Genome-wide surveys have identified 106 NAC TF members in the *Arabidopsis thaliana* (Arabidopsis hereinafter) genome, which are 149 in rice (*Oryza sativa*), 96 in cassava (*Manihot esculenta*), 167 in banana (*Musa acuminata*), 74 in grape (*Vitis vinifera*), 101 in soybean (*Glycine max*), and 163 in *Populus trichocarpa*^9–17^.

A typical NAC TF has a conserved NAC domain of ~150 amino acids at the N-terminus and a more divergent C-terminal transcription regulatory (TR) region^18,19^. The NAC domain contains five subdomains (A–E) that represent motifs for both DNA-binding (DB) and protein–protein interactions^20^. The highly conserved subdomains C and D, with net positive charges, bind to specific *cis*-regulatory DNA sequences, whereas subdomain A functions in the dimerization of the TF^18,20–22^, and the diverse subdomains B and E may be responsible for the functional diversity of the *NAC* gene^23,24^. Based on their motifs, the rice NAC proteins (OsNAC) have been classified into 15 types, from A to O, with types A–E containing five motifs in DB domains, whereas types F–O are present in NAC-like proteins with distinct motif compositions^25^. Such motif variations highlight the involvement of NAC TFs in diverse functions. The NAC domain also modulates protein binding, which may determine the fate and functions of the NAC proteins^26^. In some NACs, a highly hydrophobic region in a negative regulatory domain (NRD) may form a part of the D subunit, which suppresses transcriptional activity of the NAC TFs. The NAC-repressor domain may inhibit the activity of other TF family members such as WRKY and AP2^27^. The C-terminal TR domain may act as either an activator or a repressor, and sometimes displays protein-binding activity. In Arabidopsis, it was found that the highly divergent TR domain has 13 common motifs in 12 out of the 18 subgroups of the NAC TF^21^. In addition, an α-helical transmembrane motif (named NTLs) is present in some NACs that is required for plasma membrane or endoplasmic reticulum membrane anchoring of the C-terminal region. To date, fewer than 20 NTLs have been identified in any single plant species: 18 NTLs have been found in *A. thaliana*, 11 in soybean, and seven in maize, *Zea mays*. NTL proteins may play important regulatory roles in response to environmental cues^28–31^.

Since the cloning of the first NAC gene (*NAM*) from petunia in 1996^9^, the NAC TFs have been shown to play important roles in various biological processes, as well as responses to abiotic stresses. For example, in cotton, *Gossypium hirsutum*, the expression of some *NAC* genes such as *GhNAC22* and *GhNAC34* was strongly regulated by salinity and drought stresses, while in maize, natural variation in the *NAC* gene *ZmNAC111* was associated with drought tolerance^3,32–34^. Genome-wide survey and expression-profiling analysis further identified a set of *NAC* genes involved in adaptation to drought^35^. NAC proteins are important regulators in a wide range of developmental processes like cell wall biosynthesis, the formation of lateral roots, floral morphogenesis, development of shoot apical meristem, embryo development, and grain nutrient remobilization^36–41^.

In a recent study, we found that the expression of the cucumber gene *CUC3* encoding a NAC protein, was downregulated by >1000-fold in a spontaneous *tbh* (*tiny branched hair*) mutant as compared with its wild type, suggesting its possible involvement in cucumber fruit-trichome development^42^. In another study, a cucumber NAC gene that is a homolog of Arabidopsis *AtNAC25* showed no expression in the *tril* (*trichome-less*) glabrous mutant, but it was highly expressed in its wild type, implying that this *NAC* gene was affected by *TRIL* (*CsGL3*), a key gene for regulating trichome development^43^. In cucumber, fruit trichomes are also called spines, which together with the tubercles form the warty fruit trait^42,44^. The presence or absence, the size, number, and density of fruit spines are important fruit-quality traits affecting cucumber fruit economic value and commercial marketability^42,44,45^. For example, North China fresh market (Chinese Long) cucumbers have fruits with many trichomes and a dull surface appearance, whereas European greenhouse cucumbers often have smooth and glossy fruits (no visible warts and spines).

Despite the importance in cucumber breeding for external fruit quality, little is known about the molecular mechanisms of the initiation and development of fruit spines^44^. Although transcriptomic data from cucumber trichome-related mutants have suggested important roles of some NAC TFs in trichome organogenesis and development^42,43,46^, a systematic characterization of NAC TFs in the cucumber genome is lacking. Therefore, in this study, we performed genome-wide identification and characterization of NAC genes in the cucumber genome. To investigate the potential functions of NAC TFs in cucumber trichome development, we examined the expression of all *NAC* genes in wild-type plants and the *tbh* mutant. We identified a set of NAC genes that represent targets for future studies of cucumber fruit spine development.

## Methods

### Plant materials and hormone treatments

The cucumber inbred line R1407 (WT) and *tbh* mutant^42^ plants were grown in a greenhouse in the experimental field of the China Agricultural University (Beijing, China). Cultured practices were carried out according to recommended protocols.

To examine the effect of hormones on the expression of *CsvNAC* genes and trichome development, WT fruits (2 d before flowering) were sprayed with 5 ml of GA_3_ (50 μM), IAA (200 μM), Me-JA (200 μM), or ethephon (1 mM). In the ethephon treatment, the pots were sealed with aluminum foil to maintain the ethylene gas enriched atmosphere. Control plants were sprayed with 5 ml of H_2_O. Trichomes were collected from the fruits at 0, 6, 12, 24, and 36 h after treatment. There were three independent replicates for each treatment.

### Database search and sequence retrieval

The sequences of the 9930 cucumber draft genome (version 2.0) were extracted from the Cucurbit Genomics database (http://www.cucurbitgenomics.org/), and Arabidopsis sequences from TAIR (http://www.arabidopsis.org/). The hidden Markov model (HMM) profile of the NAM domain (PF02365) was downloaded from the Pfam database (Pfam 29.0) (http://pfam.sanger.ac.uk/) to identify *NAC* genes in the cucumber genome using HMMER 3.0 software with an E-value cutoff of 1^3^. The predicted Arabidopsis NAC protein sequences (http://www.arabidopsis.org) were used as a query against the predicted cucumber proteome sequences to identify all cucumber NAC proteins. The Multiple Expectation Maximization for Motif Elicitation (MEME) program version 4.9.1 (http://nbcr-222.ucsd.edu/opal-jobs/) was used to identify motifs in the 91 CsvNAC protein sequences. MEME was run locally with the following parameters: number of repetitions––any, maximum number of motifs––5, and the optimum motif widths were constrained to be between 6 and 250 residues. In addition, the three fields (length, molecular weight, and isoelectric point) of each NAC protein were predicted by the online program ExPasy (http://www.expasy.org/tools/).

### Phylogenetic analysis

The MEGA 5 software package (http://www.megasoftware.net) was used for the construction of unrooted phylogenetic trees using the neighbor-joining (NJ), minimal evolution (ME), and maximum parsimony (MP) methods, and bootstrap tests were carried out with 1000 replicates. The pairwise gap-deletion mode was used to ensure that the more divergent C-terminal domains could contribute to the topology of the NJ tree^3^.

### Chromosomal location and analysis of the relationship of homologous *A. thaliana* members

The nucleotide sequences of all cucumber *NAC* genes were further used as a query for NCBI BLASTn (http://blast.ncbi.nlm.nih.gov/Blast.cgi) searches against the cucumber chromosomes to determine their locations. Mapping was then conducted using the MapInspect program (http://mapinspect.software.informer.com/)^3^. Orthomcl (http://orthomcl.org/orthomcl/) was used to perform relationship analyses of homologous members between cucumber and Arabidopsis^47^. The results were formatted for display using the Circos software (version 0.69, http://circos.ca/sofware/download/circos/)^48^.

### Exon/intron structure analysis

The exon/intron arrangement of the *NAC* genes was illustrated with the gene structure display server (GSDS) program (http://gsds.cbi.pku.edu.cn/) using both genomic DNA sequences and the corresponding coding sequences. The 5′ untranslated region (UTR) sequences were removed to allow better visualization and comparison.

### RNA isolation and quantitative real-time PCR (qPCR) analysis

Roots, tendrils, male and female flowers, stems, leaves, and fruits were collected from R1407 for total RNA extraction using the Column Plant RNA out kit (TIANDZ, China) following the manufacturer’s instructions. Fruit samples were collected at −2, 0, 8, and 14 d post anthesis. Samples were treated with RNase-free DNase I (Promega, USA) and quantified spectrophotometrically. PrimeScript First-Strand cDNA Synthesis SuperMix (TaKaRa, Japan) was used for synthesis of first-strand cDNA. Primers used for qPCR were designed with Primer 5.0 and the *actin* gene was used as the internal standard in the quantitation of template cDNA^42^. The qPCR was performed using SYBR^®^ Premix Ex *Taq*^TM^ (TaKaRa, China) with the Applied Bio-systems 7500 real-time PCR system (ABI, USA). The −2 ^–ΔΔCt^ method was used to determine the relative fold differences in template abundance for each sample^42^. For semiquantitative RT-PCR, the conditions were as follows: 10 min at 94 °C, 30 s at 94 °C, 30 s at 50 °C, and 30 s at 72 °C, followed by 7 min at 72 °C. Each cycle was repeated 25 times. The RT-PCR products were separated using a 2.0% (m/v) agarose gel. The gene-specific primers are listed in Supplementary Table S1.

### *Cis*-element and miRNA target analysis

To identify putative *cis*-acting regulatory elements in the promoter regions of the *CsvNAC* genes, nucleotide sequences of the 2-kb upstream regions from the transcription start site (TSS) were retrieved from the 9930 V2.0 draft genome sequences. Promoter *cis*-element analysis was carried out using the *cis*-acting regulatory elements database (PLANTCARE) (http://bioinformatics.psb.ugent.be/webtools/plantcare/html/).

For miRNA target analysis, the mature miRNA sequences were downloaded from the miR-Base v20.0 (http://www.mirbase.org/) and RMRD (http://bioinformatics.cau.edu.cn/PMRD/). The prediction was performed using the plant small RNA target analysis server (http://plantgrn.noble.org/psRNATarget/) with default settings.

### Scanning electron microscopy (SEM) analysis

Fruits at the flowering day were treated with 1% osmium tetroxide vapor for 24 h and then air-dried for 72 h. The samples were examined by a SEM as described previously^49^. Images were collected using a Hitachi S-3400N microscope (Hitachi, Japan).

## Results

### Identification and phylogenetic analysis of NAC family members

To identify cucumber NAC TF encoding genes, the amino acid sequence corresponding to the conserved DNA-binding domain of known NAC proteins was used as the query (PF02365) in BLAST searches of the 9930 cucumber reference genome^50^. Ninety-one putative *NAC* genes were identified; each gene was annotated as *CsvNAC0XX*, where *Cs* is the genus and species initials (*Cucumis sativus*), v is the latest version (v2) of the cucumber database, and *XX* is the sequential number of a particular gene in the genome. The identified genes encoded predicted proteins ranging from 202 to 654 AA (amino acids) with isoelectric point (pI) values ranging from 4.52 to 9.53 and molecular weights from 26.2 to 73.7 kDa. Subcellular location of these genes was predicted using an online analysis tool from Molecular Bioinformatics Center (http://cello.life.nctu.edu.tw/). Among the 91 NAC proteins, one each was predicted to be located in the plasma membrane (CsvNAC019) or chloroplast (CsvNAC061); two (CsvNAC001 and 041) were extracellular; four (CsvNAC049, 53, 86, and 87) cytoplasmic; and the rest were localized in the nucleus. Detailed information, including accession numbers, exon number, sequence length, and chromosome locations of all identified NAC proteins is provided in Supplementary Table S2.

**Fig. 1:**
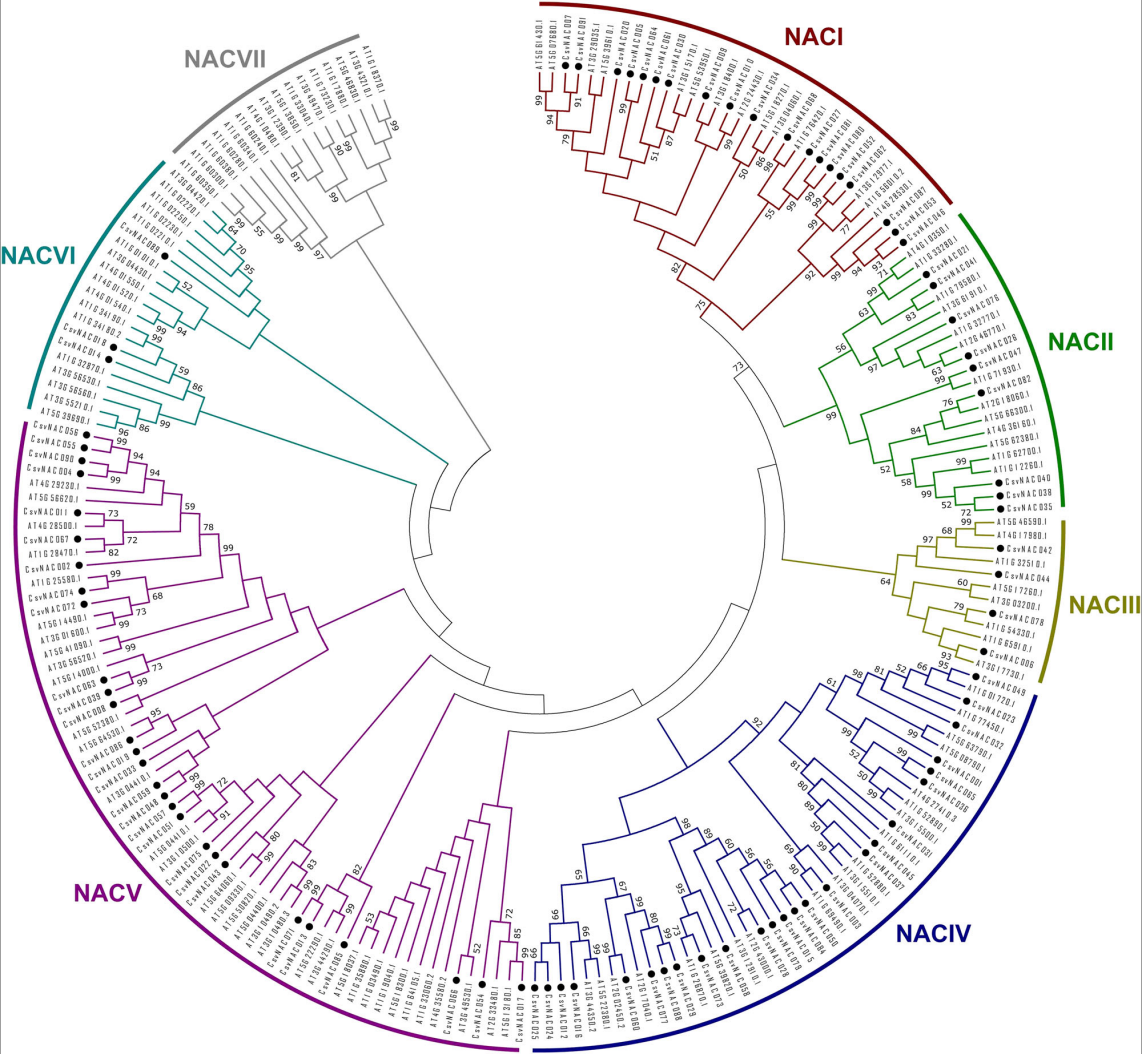
Phylogenetic tree of cucumber and *Arabidopsis thaliana* NAC proteins. The phylogenetic tree is based on sequences from NAC domain-containing proteins from cucumber (91 genes) and *A. thaliana* (121 genes). Amino acid sequences were aligned using Clustal W, and a neighbor-joining tree was constructed using MEGA 5.0 with a 1000-bootstrap replication support. Each of the seven NAC subfamilies is indicated in a specific color

In order to explore the evolutionary relationships among members of NAC TF families, a neighbor-joining tree based on cucumber and Arabidopsis NAC protein sequences was constructed (Fig. 1). All members from both species were divided into seven subgroups that were designated as NACI to NACVII, respectively. Group NACV constituted the largest clade with 28 cucumber NAC members followed by NACIV with 25 cucumber protein sequences. The smallest clade, NACVII, had no members from cucumber.

### Chromosomal locations of *CsvNAC* genes and their relationship with Arabidopsis orthologs

**Fig. 2:**
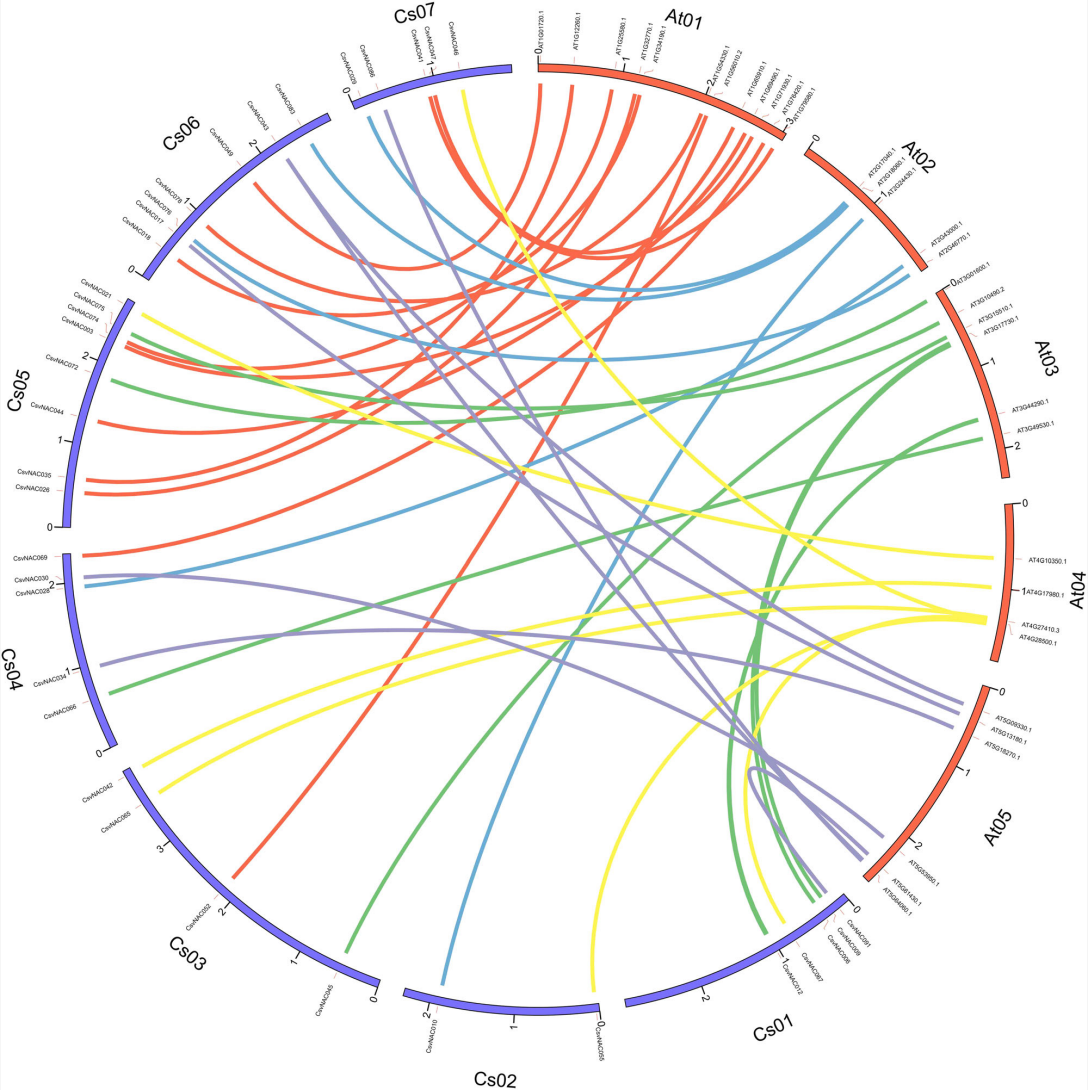
Analysis of homologous member relationships between cucumber and *A. thaliana*. Orthomcl software was used to perform homologous member relationship analysis between cucumber and *A. thaliana*. The outer blue lines show the cucumber chromosomes and the red lines represent the *A. thaliana* chromosomes. Homologous cucumber and *A. thaliana* members are connected with different colored curved lines

The 91 *NAC* genes were distributed unevenly in the cucumber genome with 14, 9, 19, 9, 11, 20, and 9 on chromosomes 1 through 7, respectively (Supplementary Figures S1 and S2). Using Orthomcl, the *CsvNAC* genes were comparatively mapped with their Arabidopsis orthologs^44^. Maximum orthology was observed between *CsvNAC* genes on cucumber chromosome 5 and Arabidopsis chromosome 1, and the *CsvNAC* genes on cucumber chromosome 6 showed 67% orthology and colinearity with the *NAC* genes on Arabidopsis chromosome 5 (Fig. 2). These results suggested similar evolutionary trends and that diversification of the NAC family occurred prior to the divergence of cucumber and Arabidopsis.

### *CsvNAC* gene structures and conserved motifs

**Fig. 3:**
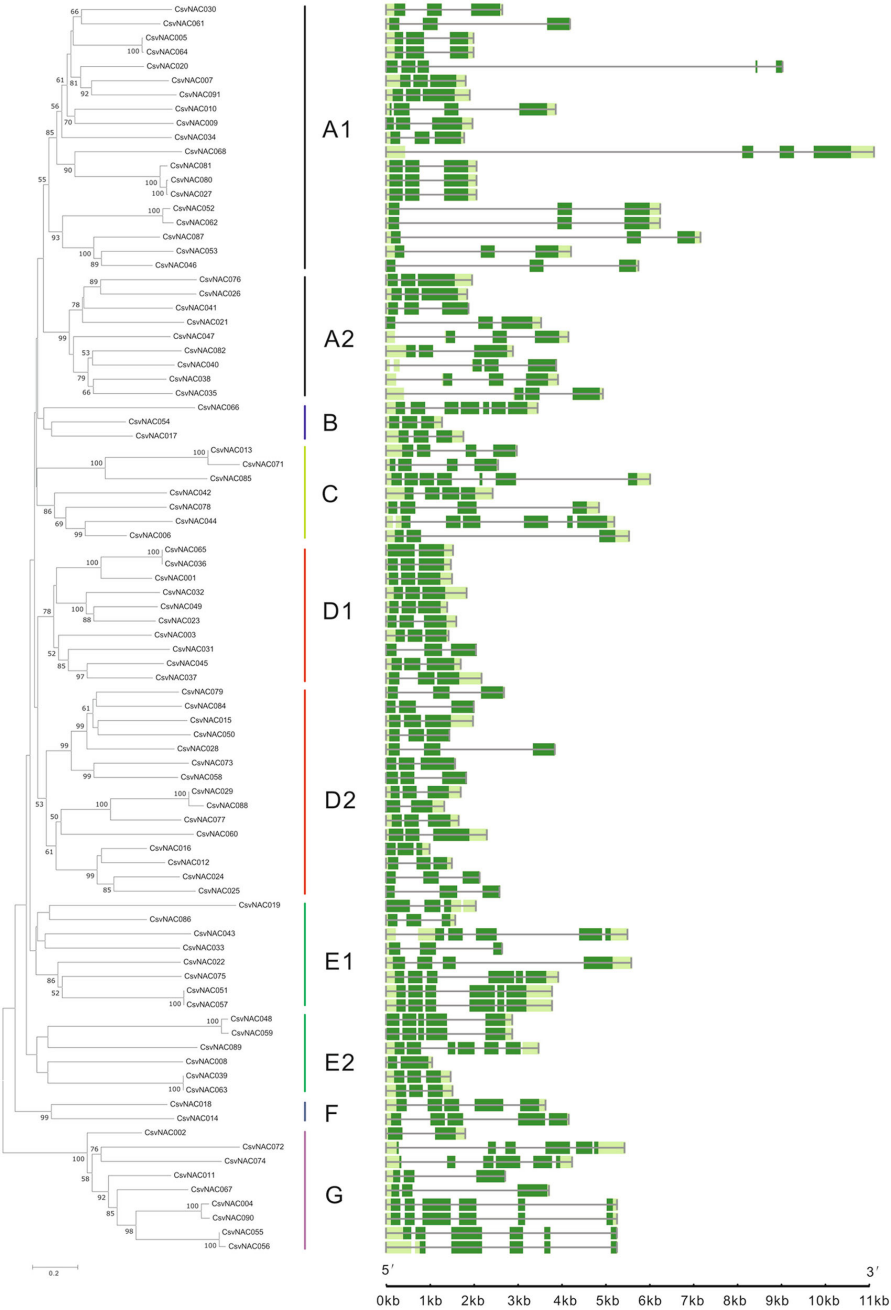
Phylogenetic analysis and exon–intron structure of the *CsvNAC* genes. **a** The phylogenetic tree was generated using the neighbor-joining (NJ) method implemented in the MEGA 5.0 software with the JTT model and the pairwise gap-deletion option. The bootstrap analysis was conducted with 1000 iterations. **b** The *CsvNAC* exon–intron distribution. The green bars indicate the exons, and the black lines indicate the introns

To better understand the relationships between the structure and functions among cucumber *NAC* genes, the exon/intron organization and conserved motifs were analyzed, which are shown in Figs. 2 and 3, respectively. In the resulting phylogenetic tree, all 91 NAC members were divided into 10 subclades (Fig. 3). It seems that the most closely related members had similar numbers of exons (Fig. 3). For example, all nine members in subgroup A2 had three exons, while the seven members in subgroup C had a varying number of 3–7 exons. Among the 26 members in groups D1 and D2, all except two had three exons (Fig. 3).

**Fig. 4:**
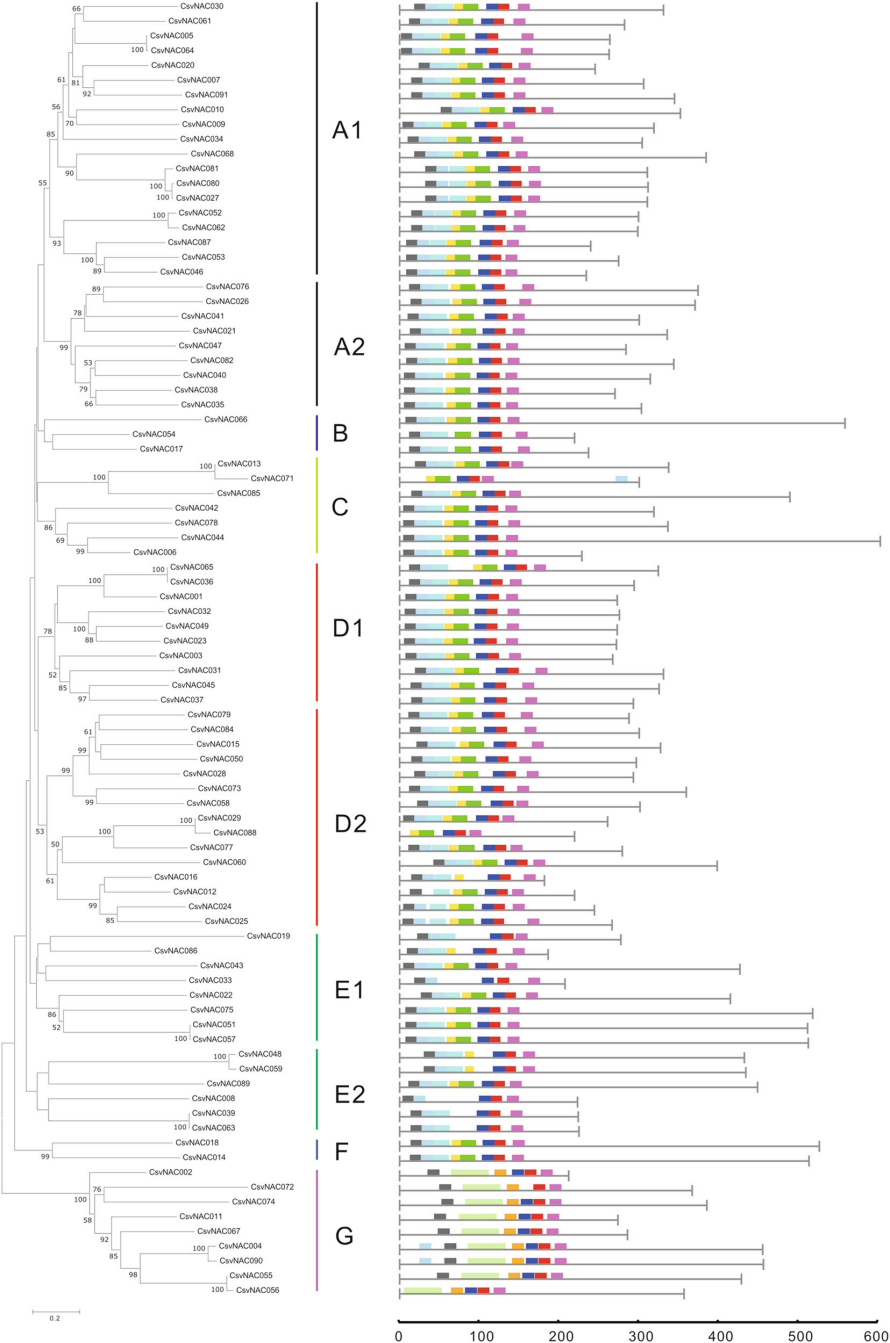
Phylogenetic analysis and conserved motifs of the *CsvNAC* genes. **a** The phylogenetic tree was generated using the Neighbor-Joining (NJ) method implemented with MEGA 5.0 software and the pairwise gap-deletion option. The bootstrap analysis was conducted with 1000 iterations. **b** The motif composition of the cucumber *NAC* genes. Each color represents a specific motif

Ten conserved motifs were identified among the 91 CsvNAC proteins (Supplementary Figure S3). As expected, the most closely related members in the same subfamilies shared a common motif composition, which may be indicative of similar functions (Fig. 4). Motif 7 (blue box in Fig. 4) was observed in all CsvNAC proteins, and most of the predicted motifs were located at the N-terminus, which contains the A–E subdomains (motif 1–7 and motif 9) that confer DNA-binding activity. These results are consistent with previous studies showing a connection between subfamilies and motifs^22^.

### *Cis*-element analysis

Many *cis*-elements were detected in the *CsvNAC* gene promoter regions including some involved in hormone signaling, such as auxin, ethylene (Eth), gibberellin (GA), abscisic acid (ABA), and salicylic acid (SA). Most of the cucumber *CsvNAC* promoters contained the conserved CGTCA element, suggesting self-regulation, since this is a sequence known to be bound by NAC TFs^35^. Some promoters also contained several abiotic stress response elements. For instance, *CsvNAC053*, *085*, and *088* had more than one EDR or LRT element, which are known to be related to drought and chilling stresses^31^. In six NAC genes (*CsvNAC010*, *041*, *048*, *053*, *058*, and *059*), *cis*-element analysis also revealed HD-Zip I binding sites in their promoters, which are important for trichome development^42,43^. Additional information regarding *cis*-acting regulatory elements that are involved in meristem specific activation, methyl jasmonate (Me-JA)-responsiveness or differentiation of the palisade mesophyll cells, is provided in Supplementary Table S3.

### *CsvNAC* expression profiles in different organs in wild type and *tbh* mutant plants

**Fig. 5:**
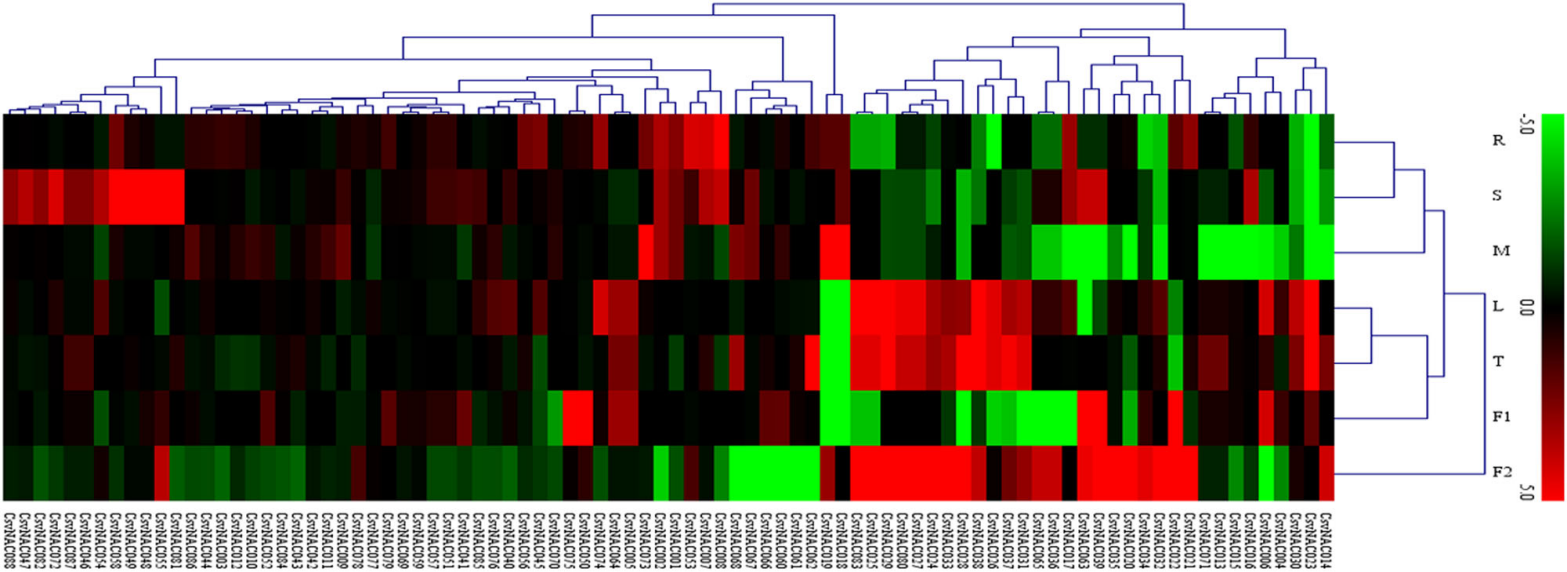
Expression profiles of the *CsvNACs* among different tissues under normal conditions in R1407 (WT). The heat map was generated by MultiExperiment Viewer v4.2 software^3^. The expression data were gene-wise normalized and hierarchical clustered with average linkage. Each row corresponds to the relative expression levels normalized against the maximum value. The bar at the top of the heat map indicates relative expression values; thereby, values 5, 0, −5 represent high, intermediated and low expression, respectively. The tissues of cucumber are indicated by the abbreviations: Root (R), Stem (S), Tendril (T), Male flower (M), Female flower (F1), Fruit (8 d after anthesis) (F2). Red stands for relative high gene expression level and green stands for relative low gene expression level. Three independent replicates were used for each sample

To gain insight into the temporal and spatial transcription patterns and putative functions of the *CsvNAC* genes in cucumber growth and development, we examined the expression patterns of all 91 *NAC* genes in the root, stem, leaf, tendril, male flower, female flower, and fruit with qRT-PCR (Fig. 5). Organ specific expression of different *NAC* genes was obvious. For example, 11 genes (*CsvNAC047, 048, 049, 054, 055, 058, 071, 072, 081, 082*, and *088*) were highly expressed in the stem, while 12 (*CsvNAC020, 021, 022, 024, 027, 028, 029, 032, 033, 035, 038*, and *078*) were specifically expressed in the fruit. Similarly, small clusters of genes were specifically expressed in female flowers or leaves (Fig. 5, Supplementary Table S4).

**Fig. 6:**
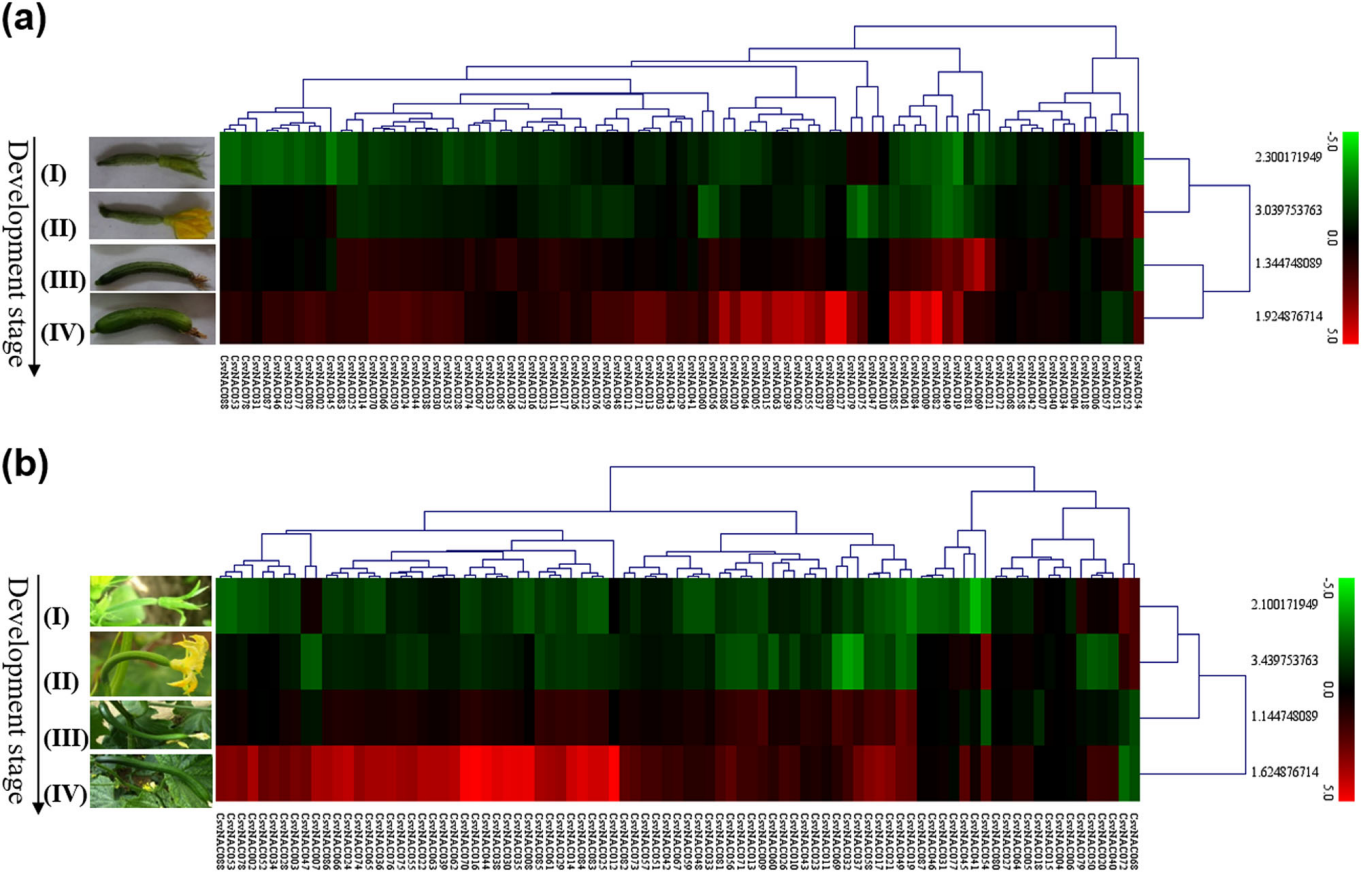
Expression profiles of CsvNACs in four developmental stages in cucumber lines. Expression profiles of *CsvNACs* in different fruit developmental stages in R1407 (**a**) and the *tbh* mutant (**b**). The heat map was generated by Multi Experiment Viewer v4.2 software^3^. The expression data were gene-wise normalized and hierarchical clustered with average linkage. Each row corresponds to, the relative expression levels normalized against the maximum value. The bar on the right of the heat map indicates relative expression values; thereby, values 5, 0, and −5 represent upregulated, unaltered, and downregulated expression, respectively. Four development stages on the left of cucumber fruits were used in this study: (I) 2 d before flowering (DAF); (II) The day of flowering; (III) 8 d DAF; and (IV) 14 d DAF. Red stands for relative high gene expression level and green stands for relative low gene expression level. Three independent replicates were used for experiment

We examined expression of *CsvNAC* genes in fruits at four development stages of both wild-type cucumber (R1407) and the *tbh* mutant (Fig. 6, Supplementary Table S5). Except for *CsvNAC003, 045, 047, 051*, *054*, *057*, *072, 075*, and *079*, most other *CsvNAC* genes exhibited similar expression profiles at Stages I (2 d before flowering) and II (The day of anthesis) (Fig. 6). It is noted that at stage III (8 d after flowering). Interestingly, *CsvNAC069* exhibited high and slightly high expression in R1407 and the *tbh* mutant, respectively only at the 8^th^ day post anthesis. Moreover, the expression level of other two genes, *CsvNAC081* and *CsvNAC049* were obvious increased at Stage III in R1407. One cluster of *CsvNA*C genes showed dramatic increase in their expression at stage IV, which was 14 d post anthesis. Since the spines were well developed by this time^42^, likely, they do not have any direct link with spine initiation or development (Supplementary Table S5).

### *CsvNAC* gene expression during trichome development in response to hormone treatments

**Fig. 7:**
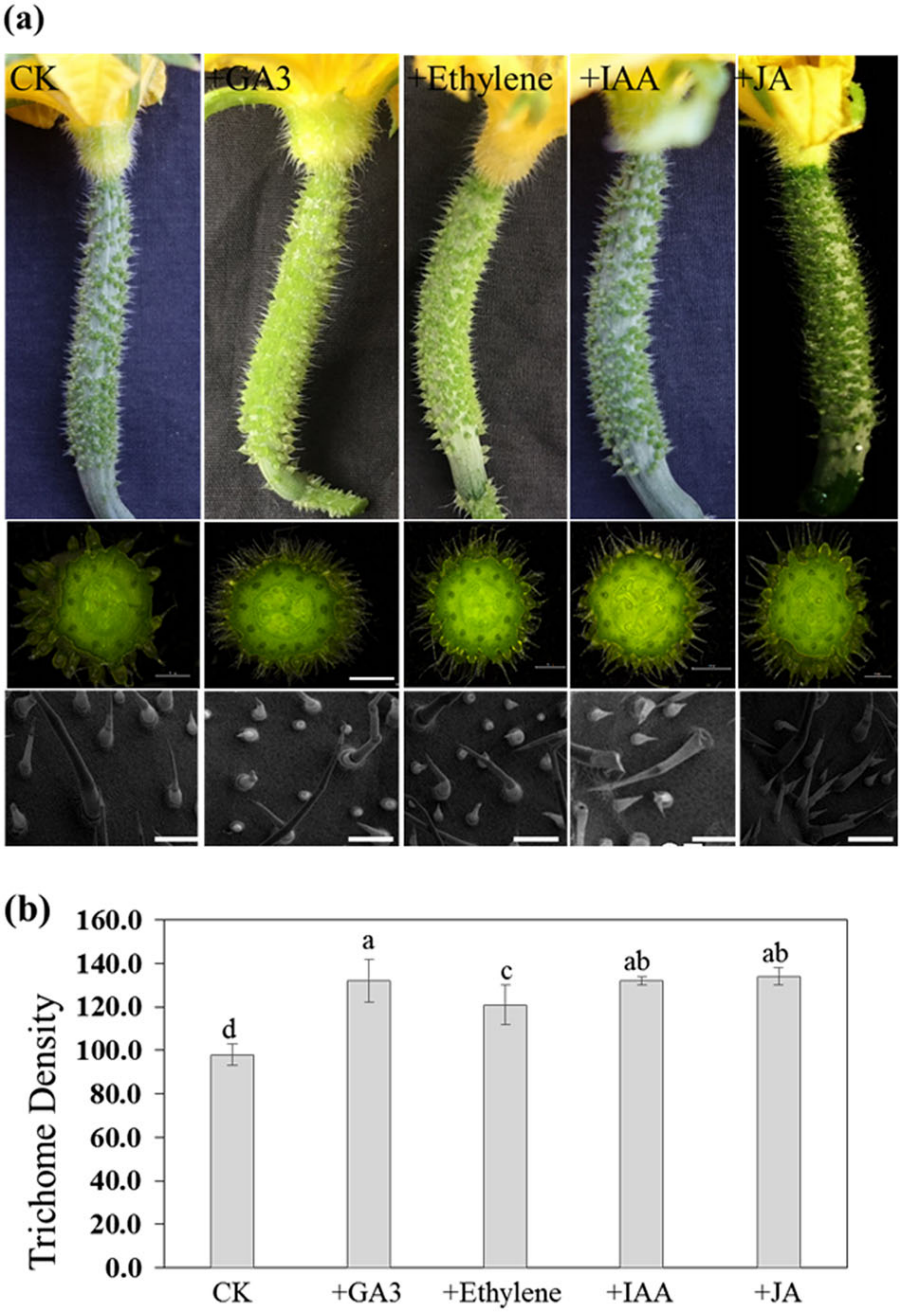
Hormone treatments increase trichome numbers in cucumber fruit. **a** Fruits were either grown for 3 d under control treatment (wt) or for 3 d under 50 μM GA3, 1 mM ethylene, 200 μM IAA, and 200 μM Me-JA. Scanning electron micrograph of trichomes stimulated after hormone treatments, showing a high density of trichomes. Scale bar = 200μm. **b** Trichome numbers vary more in hormone treated than in those grown in the control. For each treatment, 20 whole fruit were examined. Mean trichome numbers (±SD) are shown

**Fig. 8:**
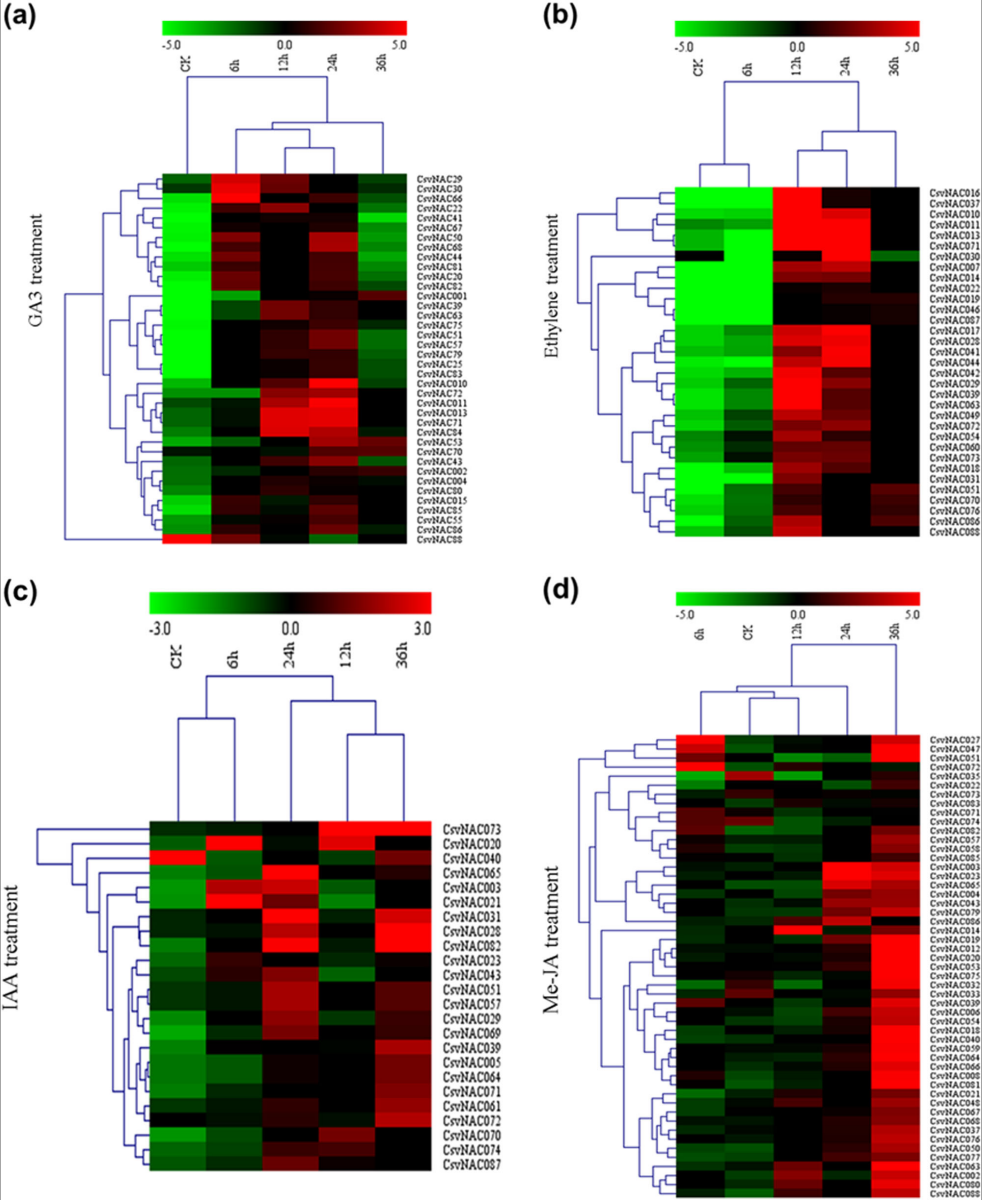
Expression profiles of *CsvNAC* transcription following hormone treatments with Gibberellin (GA3), Ethylene (Eth), Auxin (IAA) and Jasmonic acid (Me-JA) in R1407. The heat map was generated by Multi Experiment Viewer v4.2 software^3^. The expression data were gene-wise normalized and hierarchical clustered with average linkage. Each row corresponds to, the relative expression levels normalized against the maximum value. The bar on the right of the heat map indicates relative expression values; thereby, values 5, 0, and −5 represent upregulated, unaltered, and downregulated expression in (**a**,**b**,**d**), respectively; 3,0, and−3 show high, intermediate, and low expression in (**c**), respectively. The *CsvNAC* genes are listed on the right of the expression array under GA3 (**a**), Ethylene (**b**), IAA(**c**), and Me-JA (**d**). Red stands for relative high gene expression level and green stands for relative low gene expression level. Three independent replicates were used for this experiment

The effects of phytohormones, such as GA, IAA, Me-JA, and Eth, on trichome proliferation and development have been well documented^26,51^. To understand the effects of hormones on trichome development, we examined trichome density dynamics in response to treatments with GA, IAA, Me-JA, and Eth. We found that application of these hormones stimulated trichome formation and increased trichome density on the fruits (Fig. 7a). The spine density on treated fruits was significantly higher than that in the control (Fig. 7b) suggesting GA, IAA, Eth, and Me-JA may all promote trichome initiation. To further determine whether *CsvNAC* genes responded to hormone related signaling, *cis*-element analysis in the 2 kb promoter region of each gene was conducted (Supplementary Table S6). Motifs related with GA, IAA, Me-JA, and Eth in the promoters were identified and their corresponding transcript abundance was measured following treatments with different hormones. Application of all phytohormones significantly upregulated the expression of *NAC* genes in fruit spines (Fig. 8). For example, upon GA3 application, the expression of most *CsvNAC* genes was upregulated peaking at 6–24 h post-treatment, followed by a sharp decline to the pre-treatment level, or to a level that was lower than in the control (Fig. 8a, Supplementary Table S7). Similarly, a substantial increase in the expression of all selected *CsvNAC* genes was observed 12 or 24 h post-treatment with Eth, with the exception of *CsvNAC30*, *031*, *054*, *070*, and *088*, all of which were downregulated (Fig. 8b, Supplementary Table S7). However, none of these genes showed a continuing elevated expression after 24 h of treatment. In a few cases, changes in the expression were observed following treatment with IAA. For example, *CsvNAC020* and *CsvNAC051* showed their highest transcript levels at 6 h post-application, and *CsvNAC065* reached its peak of expression at 24 h after treatment. In general, a slight increase in expression of selected genes was observed 24 h after treatment (Fig. 8c, Supplementary Table S7). Me-JA induced expression of most *CsvNAC* genes after 24–36 h treatment, with the exception of *CsvNAC002* and *CsvNAC074* (Fig. 8d, Supplementary Table S7). In contrast, *CsvNAC032*, *CsvNAC033*, and *CsvNAC035* showed decreased transcript levels 6–12 h treatment, while the expression of *CsvNAC072* and *CsvNAC071* peaked at 6 h post Me-JA treatment and then declined. Taken together, exogenous hormones may affect *CsvNACs* expression so as to regulate trichome formation in cucumber.

### *CsvNAC* miRNA targets and gene ontology (GO) annotation

Micro-RNAs (miRNAs) play roles in post-transcriptional gene regulation by either cleaving mRNA transcripts or repressing translation^41^. To assess the involvement of miRNAs in regulating the expression of *CsvNAC* genes, putative miRNA targets were determined in the 91 *CsvNAC* genes. Twelve were found to be the targets of 13 known cucumber miRNAs (Supplementary Table S6 and Supplementary Table S8). *CsvNAC091* and *CsvNAC075* were each predicted to be the targets of 2 miRNAs (miR-164c and miR-164e-5p, or miR-169 and miR-169n, respectively). MiR-164a was predicted to target *CsvNAC064*, *CsvNAC020*, and *CsvNAC005*, and miR-164b to target *CsvNAC062*, *CsvNAC061*, and *CsvNAC052*. These *CsvNAC* targets belonged to Subclass A1. In order to better understand the characteristics of these miRNA targets, GO enrichment analysis was performed using Blast2Go (https://www.blast2go.com/). This analysis suggested participation of the 12 CsvNAC proteins in diverse biological processes (Supplementary Table S8). Of the 4 categories of biological processes defined by Blast2Go, the CsvNAC proteins were predicted to function predominantly in ‘developmental processes’ (75%) (Supplementary Table S8), followed by ‘multicellular organismal processes’ and ‘cellular processes’. Cellular component prediction further suggested that 10 out of the 12 proteins are localized to the nucleus, and molecular function analysis suggested that those NAC proteins have nucleic acid binding TF activity (Supplementary Table S8).

## Discussion

In this study, we performed a comprehensive analysis of the cucumber *CsvNAC* family genes to examine their potential functions in fruit-trichome development. The 91 full-length NAC family proteins could be clustered into 6 subfamilies (NACI –VI), which was in contrast with 7 of Arabidopsis NAC proteins; no CsvNAC members were in the small NACVII clade with 16 Arabidopsis NAC members (Fig. 1). This may suggest functional divergence of some NAC genes between the two species. There were significantly more exons in members of C and G subfamilies (4–7) than in the D1 and D2 subfamilies (2–3). It has previously been reported that the rate of intron loss is faster than the rate of intron gain after segmental duplication in rice NAC genes^52^. Thus, it is likely that subfamilies C and G may contain more primitive genes, from which NAC genes in other clusters were derived. This phenomenon has also been reported in other species, such as Vitis vinifera and Manihot esculenta^53^. Conserved motif analysis revealed that almost all the CsvNAC proteins contained canonical A to E subdomains. In addition, each subfamily had other common motifs, while some subfamilies also harbored specific motifs (Fig. 4). Consistent with studies in Arabidopsis, rice, grapevine and cassava, our results suggest that CsvNAC TFs are evolutionarily conserved^16–19,53,54^.

To identify NAC genes playing possible roles in fruit spine development, we examined *CsvNAC* gene expression in ovaries/fruits from 2 d before flowering to 14 d post anthesis in R1407 and the spontaneous *tbh* trichome mutant (Fig. 6). We identified 12 *CsvNAC* genes differentially expressed in the *tbh* mutant and R1407 from stages I to III. Based on our previous work, stages I and II are corresponding to spine initiation and formation; fruit spines mature at stage III^42^. Six genes, *CsvNAC051*, *057*,*003*, *047*, *075*, and *045* showed higher expression in the wild type (R1407) than in the *tbh* mutant suggesting they may play positive regulatory roles in fruit spine development (Fig. 6). The expression pattern of some of these genes was consistent with previous studies^42^. For example, *CsvNAC003* was not expressed in the glabrous *tril* mutant, but highly expressed in the wild-type background, suggesting its involvement in trichome development^43^. In the present study, this gene also exhibited decreased expression in the *tbh* mutant as compared with the WT (Fig. 6). It’s homolog genes in cotton, *GhNAC31, 49, 73*, and *77* exhibit significantly higher expression at 10 d post anthesis (DPA) and were proposed to be involved in the early elongation phase of fiber development^3^. On the other hand, the three genes, *CsvNAC079*, *CsvNAC072*, and *CsvNAC054* were highly expressed in *tbh* at stages I and II indicating their possible negative regulatory roles in spine development. It is worth to note that *CsvNAC069* had and low expression in R1407 and in the tbh mutant only at the 8^th^ day post anthesis, which may suggest that *CsvNAC069* plays its role in spine maturation. A cluster genes showed differential expression at stage IV, which may indicate their involvement in senescence of spines^34^. To summarize, our expression profiling of these *CsvNAC* genes provide us a global picture on their roles in spine initiation and development.

Plant hormones such as GA, Me-JA, and IAA are also known to influence trichome development^51,54,55^. A recent study found that GA distribution in *Arabidopsis* leaf mesophyll affected epidermis cell fate specification and promoted trichome formation^55^. Me-JA and its derivatives function as key signaling molecules in trichome formation; exogenous Me-JA causes an increase in the number of leaf trichomes in Arabidopsis^51^. The auxin response factor (*ARF*) genes are involved in auxin (IAA) signaling transduction. In *SlARF3* RNA interference (RNAi) lines in tomato, *SlARF3*-downregulated plants exhibited reduced density of types I, V, and VII trichomes on the leaves, which indicated the important roles of IAA transduction in the formation of trichomes in tomato^54^. Notably, we also found application of those hormones stimulated trichome formation and increased trichome density on the fruits (Fig. 7), suggesting GA, IAA, and Me-JA may all promote trichome initiation. Previous work focused on plant leaf trichomes^51,55^. Here we found that GA, IAA, and Me-JA also play a vital role on the cucumber fruit spine development. In response to treatments with 4 hormones ost *CsvNAC* genes showed first increased then decreased expression pattern (Fig. 8), suggesting that most of these *CsvNAC* genes might have GA, Eth, IAA, and Me-JA responsive elements, which may be due to crosstalks among the four hormone signaling pathways. However, some other genes, (e.g., *CsvNAC007* and *CsvNAC032*) responded specifically to treatments with Eth and JA, respectively, indicating they may contain Eth and JA response elements and involvement of Eth and JA in inducing trichome development through the regulation of these TFs (Fig. 8). Indeed, most cucumber *CsvNAC* gene promoters contained the conserved AAACAGA (Gibberellin-responsive), CGTCA (Me-JA responsive), ATTTCAAA (Ethylene-responsive), and AACGAC (Auxin-responsive) elements (Supplementary Table S3), which can explain why treatments with these hormones caused *CsvNACs* transcriptional changes. Thus, these data suggested a connection among *CsvNACs* function, hormones signaling and spine development.

miRNAs are known to be involved in many aspects of plant development, but there were few reports on NAC miRNA targets specifically involved in plant trichome development. Here we found 12 *CsvNAC* genes that were potentially the targets of 13 miRNAs (Supplementary Table S6 and Supplementary Table S8). Most of the targets belonged to subclass A1, in which several trichome development related genes are located, indicating that the miRNAs identified here may be helpful in better understanding the transcriptional regulation of genes involved in this process. GO analysis further indicated that most of the target genes are involved in developmental processes (Supplementary Table S8), followed by multicellular organismal processes.

In summary, we identified 91 *NAC* genes in the cucumber genomes and examined their key structural features. The phylogenetic relationships among these *NAC* genes were consistent with their exon/intron structurem, as well as the distribution of conserved domains. We identified tissue-specific, fruit development-dependent differentially expressed *CsvNAC* genes in the *tbh* mutant, and its wild-type R1407 cucumber. Most genes were responsive to treatments by 4 hormones. A significant portion of the *CsvNAC* genes displayed preferential expression in fruit spines. Moreover, we identified 13 known cucumber miRNAs that target *CsvNACs*. The information obtained from this study provides new insights into potentials roles of *CsNAC* genes in regulating cucumber fruit spine development. Those differentially expressed genes and miRNAs predicted from this analysis should be very valuable for functional analysis of *CsNAC* genes. This work also has practical value in developing cultivars adapting to different market requirements.

## Electronic supplementary material

Supplementary Figure S1

Supplementary Figure S2

Supplementary Figure S3

Supplementary Figure S4

Supplementary Figure S5

Supplementary Table S1

Supplementary Table S2

Supplementary Table S3

Supplementary Table S4

Supplementary Table S5

Supplementary Table S6

Supplementary Table S7

Supplementary Table S8
