# Supplementary figures and images for: Comprehensive analysis of NAC transcription factors and their expression during fruit spine development in cucumber (Cucumis sativus L.)
Source: Hortic Res. 2018 Jun 1;5:31. doi: 10.1038/s41438-018-0036-z (PMC5981648; doi:10.1038/s41438-018-0036-z)

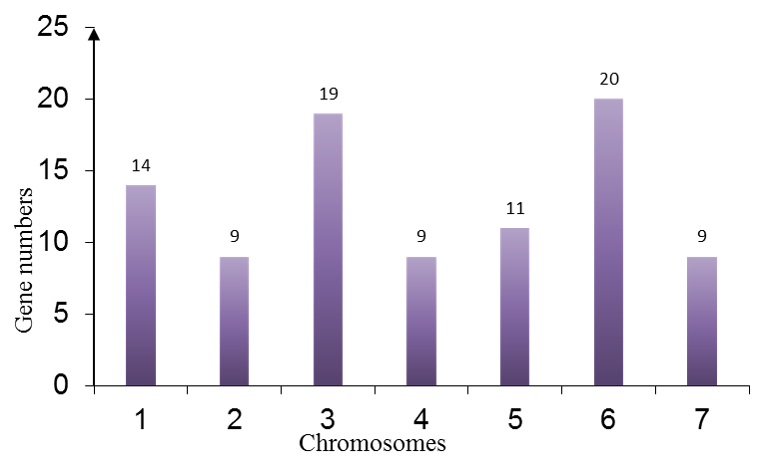

Supplement: Supplementary file 2 — Supplementary Figure S2 [file 41438_2018_36_MOESM2_ESM.tif]

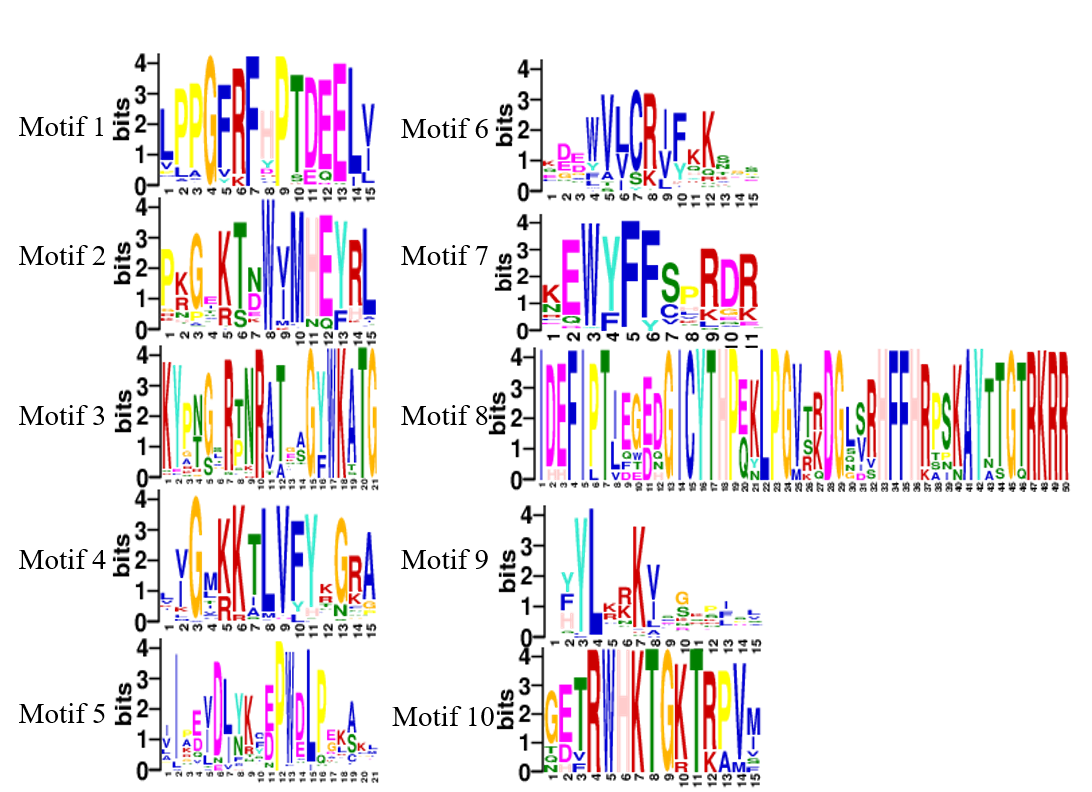

Supplement: Supplementary file 3 — Supplementary Figure S3 [file 41438_2018_36_MOESM3_ESM.tif]

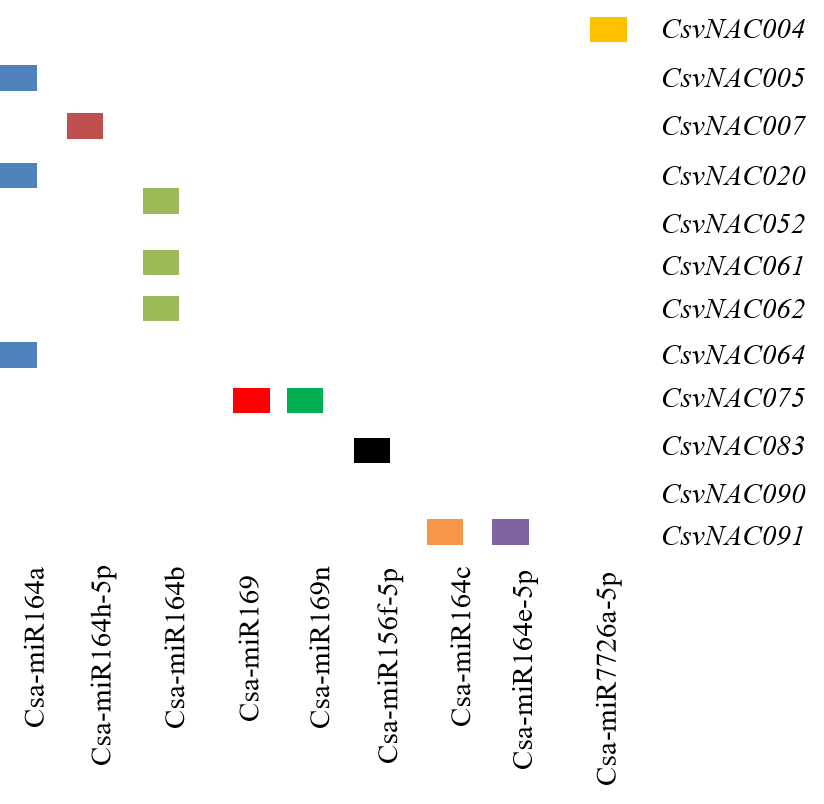

Supplement: Supplementary file 4 — Supplementary Figure S4 [file 41438_2018_36_MOESM4_ESM.tif]

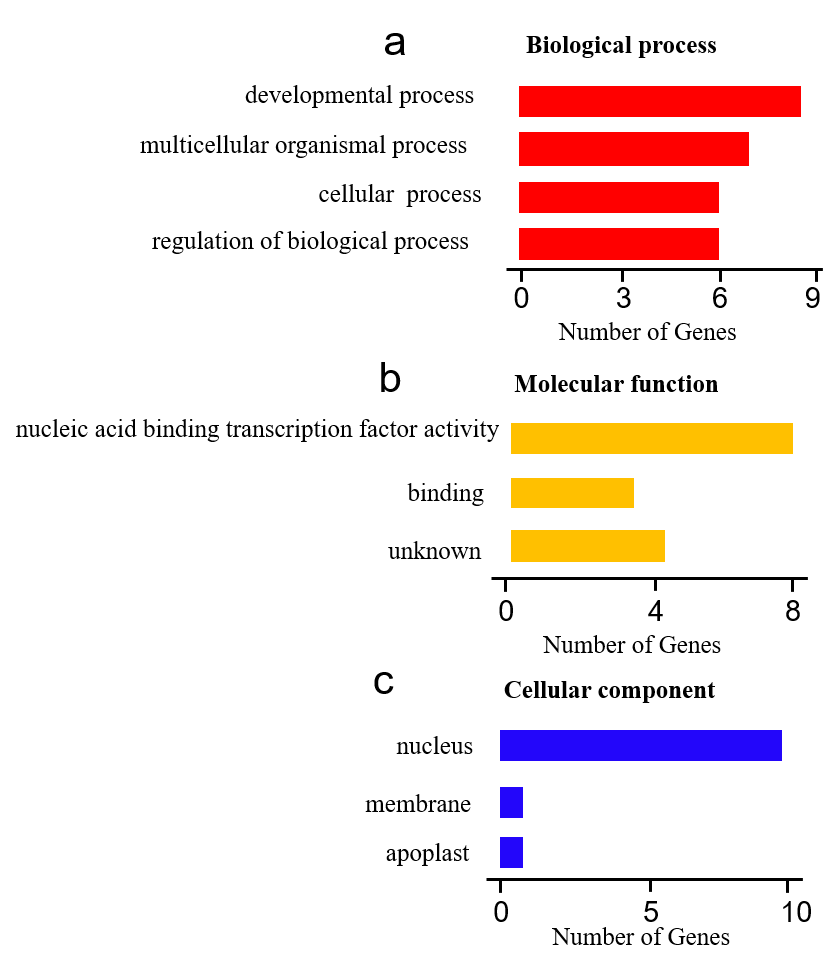

Supplement: Supplementary file 5 — Supplementary Figure S5 [file 41438_2018_36_MOESM5_ESM.tif]
